# Supplementary material for: Mitochondria delay action potential propagation
Source: Commun Biol. 2025 Sep 9;8:1341. doi: 10.1038/s42003-025-08583-x (PMC12420829; doi:10.1038/s42003-025-08583-x)
Supplement: Supplementary file 3 — Reporting summary [file 42003_2025_8583_MOESM3_ESM.pdf]

nature portfolio

Corresponding author(s): Pepe Alcami

Last updated by author(s): Jun 16, 2025

# Reporting Summary

Nature Portfolio wishes to improve the reproducibility of the work that we publish. This form provides structure for consistency and transparency in reporting. For further information on Nature Portfolio policies, see our Editorial Policies and the Editorial Policy Checklist.

## Statistics

For all statistical analyses, confirm that the following items are present in the figure legend, table legend, main text, or Methods section.

| n/a | Confirmed | |
|---|---|---|
| ☐ | ☒ | The exact sample size (*n*) for each experimental group/condition, given as a discrete number and unit of measurement |
| ☐ | ☒ | A statement on whether measurements were taken from distinct samples or whether the same sample was measured repeatedly |
| ☐ | ☒ | The statistical test(s) used AND whether they are one- or two-sided<br>*Only common tests should be described solely by name; describe more complex techniques in the Methods section.* |
| ☒ | ☐ | A description of all covariates tested |
| ☒ | ☐ | A description of any assumptions or corrections, such as tests of normality and adjustment for multiple comparisons |
| ☐ | ☒ | A full description of the statistical parameters including central tendency (e.g. means) or other basic estimates (e.g. regression coefficient) AND variation (e.g. standard deviation) or associated estimates of uncertainty (e.g. confidence intervals) |
| ☐ | ☒ | For null hypothesis testing, the test statistic (e.g. *F*, *t*, *r*) with confidence intervals, effect sizes, degrees of freedom and *P* value noted<br>*Give P values as exact values whenever suitable.* |
| ☒ | ☐ | For Bayesian analysis, information on the choice of priors and Markov chain Monte Carlo settings |
| ☒ | ☐ | For hierarchical and complex designs, identification of the appropriate level for tests and full reporting of outcomes |
| ☐ | ☒ | Estimates of effect sizes (e.g. Cohen's *d*, Pearson's *r*), indicating how they were calculated |

*Our web collection on statistics for biologists contains articles on many of the points above.*

## Software and code

Policy information about availability of computer code

| | |
|---|---|
| Data collection | Electron microscopy images were collected using a Gatan DigitalMicrograph software (version 2.3).<br>Light microscopy images were acquired with a NIS-Elements software (version 5.02.00). |
| Data analysis | Image analysis was performed using ImageJ. Data was analyzed using Igor Pro software (version 8.0) for plotting results and both Igor Pro software (version 8.0) and R (version 4.4.1) for statistical analyses. Modeling results were analyzed using Neuron software (version 7.4) and Igor Pro software (version 8.0). |

For manuscripts utilizing custom algorithms or software that are central to the research but not yet described in published literature, software must be made available to editors and reviewers. We strongly encourage code deposition in a community repository (e.g. GitHub). See the Nature Portfolio guidelines for submitting code & software for further information.

## Data

Policy information about availability of data

All manuscripts must include a data availability statement. This statement should provide the following information, where applicable:

- Accession codes, unique identifiers, or web links for publicly available datasets
- A description of any restrictions on data availability
- For clinical datasets or third party data, please ensure that the statement adheres to our policy

Source data for this study are available in the Supplementary Source Data File. Original data is available from the corresponding author upon reasonable request.

The NEURON model for mitochondria containing axons used to generate the data for Figure 3 will be available from ModelDB upon publication (https://modeldb.science)

## Human research participants

Policy information about studies involving human research participants and Sex and Gender in Research.

| | |
|---|---|
| Reporting on sex and gender | NA |
| Population characteristics | NA |
| Recruitment | NA |
| Ethics oversight | NA |

Note that full information on the approval of the study protocol must also be provided in the manuscript.

# Field-specific reporting

Please select the one below that is the best fit for your research. If you are not sure, read the appropriate sections before making your selection.

☒ Life sciences ☐ Behavioural & social sciences ☐ Ecological, evolutionary & environmental sciences

For a reference copy of the document with all sections, see nature.com/documents/nr-reporting-summary-flat.pdf

# Life sciences study design

All studies must disclose on these points even when the disclosure is negative.

| | |
|---|---|
| Sample size | We quantified mitochondria distributions in n = 69 axons in n = 2 animals, which were sufficient to estimate the range of values used for modeling. |
| Data exclusions | In three axon cross sections we found two to three mitochondria. These were excluded from the mitochondria to axon ratio calculations based on the ellipse fits, and the correlation of axon diameters and mitochondria diameters as reported in the Methods. |
| Replication | The measurements were similar across animals. |
| Randomization | Our study does not divide animals into experimental and control groups. |
| Blinding | Investigators were not blinded to group allocation since animals were not allocated to experimental groups. |

# Reporting for specific materials, systems and methods

We require information from authors about some types of materials, experimental systems and methods used in many studies. Here, indicate whether each material, system or method listed is relevant to your study. If you are not sure if a list item applies to your research, read the appropriate section before selecting a response.

## Materials & experimental systems

| n/a | Involved in the study | |
|---|---|---|
| ☐ | ☒ | Antibodies |
| ☒ | ☐ | Eukaryotic cell lines |
| ☒ | ☐ | Palaeontology and archaeology |
| ☐ | ☒ | Animals and other organisms |
| ☒ | ☐ | Clinical data |
| ☒ | ☐ | Dual use research of concern |

## Methods

| n/a | Involved in the study | |
|---|---|---|
| ☒ | ☐ | ChIP-seq |
| ☒ | ☐ | Flow cytometry |
| ☒ | ☐ | MRI-based neuroimaging |

## Antibodies

| | |
|---|---|
| Antibodies used | The antibody 3A10 (Developmental Studies Hybridoma Bank) was used to visualize axons in the motor pathway between song nuclei HVC and RA. |
| Validation | The antibody 3A10 generated in mice (host species) recognizes a neurofilament-associated antigen from chicken. The monoclonal |

| | |
|---|---|
| Validation | antibody has been shown to label axons in avian and non-avian species (e.g. in chicken: Datar et al. The roles of microtubules and membrane tension in axonal beading, retraction, and atrophy. Biophysical Journal 117, 880-891, September 3, 2019, doi: 10.1016/j.bpj.2019.07.046). |

## Animals and other research organisms

Policy information about studies involving animals; ARRIVE guidelines recommended for reporting animal research, and Sex and Gender in Research

| | |
|---|---|
| Laboratory animals | Adult domesticated canaries (Serinus canaria). |
| Wild animals | This study did not involve wild animals. |
| Reporting on sex | Males were used in this study, where we investigated the neural pathway for song production, since singing is a sex-specific behavior restricted to males in this species. |
| Field-collected samples | This study did not involve any samples from the field. |
| Ethics oversight | Housing, welfare of the animals and experimental procedures complied with the requirements of the European Directive on the protection of animals used for scientific purposes 2010/63/EU of the European parliament and the German Animal Protection Act. |

Note that full information on the approval of the study protocol must also be provided in the manuscript.
